# Pathway to psychiatric care in Japan: A multicenter observational study

**DOI:** 10.1186/1752-4458-2-14

**Published:** 2008-09-26

**Authors:** Daisuke Fujisawa, Naoki Hashimoto, Yayoi Masamune-Koizumi, Kotaro Otsuka, Masaru Tateno, Gaku Okugawa, Atsuo Nakagawa, Ryoko Sato, Toshiaki Kikuchi, Eita Tonai, Kosuke Yoshida, Takatoshi Mori, Hidehiko Takahashi, Soichiro Sato, Hiroyasu Igimi, Yoshibumi Waseda, Takefumi Ueno, Ippei Morokuma, Katsuyoshi Takahashi, Norman Sartorius

**Affiliations:** 1 Japan Young Psychiatrists Organization Pathway Study Group, 1-23-23-7F Ebisu, Shibuya-ku, Tokyo, Japan; 2 Department of Neuropsychiatry, Keio University School of Medicine, 35 Shinanomachi, Shinjuku-ku, Tokyo, Japan; 3 Division of Psychiatry, Sakuragaoka Memorial Hospital, 1-1-1 Renkoji, Tama-shi, Tokyo, Japan; 4 Department of Psychiatry, School of Medicine, Hokkaido University, North15, West7, Sapporo, Japan; 5 Psychiatry Division, Kodama Hospital, 2-5-7 Shimomachi, Ishinomaki, Miyagi, Japan; 6 Department of Neuropsychiatry, Iwate Medical University, 19-1 Uchimaru, Morioka-shi, Iwate, Japan; 7 Division of Neuropsychiatry, Sapporo Medical University, South 1, West16, Chuo-ku, Sapporo, Hokkaido, Japan; 8 Department of Neuropsychiatry, Kansai Medical School,10-15 Fumizonomachi, Moriguchi-shi, Osaka, Japan; 9 Department of Psychiatry, Yokohama City University, 3-9 Fukuura, Kanazawa-ku, Yokohama, Kanagawa, Japan; 10 Department of Psychiatry, School of Medicine Fukuoka University, 7-45-1 Nanakuma, Jonan-ku, Fukuoka, Japan; 11 Division of Psychiatry, Takagi Hospital, 1165 Nakanomachihei, Shimabara-shi, Nagasaki, Japan; 12 National Institute of Radiological Sciences, 4-9-1 Anakawa, Inage-ku, Chiba, Japan; 13 Division of Psychiatry, Zikei Hospital, 100-2 Urayasuhonmachi, Okayama-shi, Okayama, Japan; 14 Department of Neuropsychiatry, Kurume University School of Medicine, 281 Shiromaru Oaza Buzen-shi, Fukuoka, Japan; 15 Department of Neuropsychiatry, Kyushu University Graduate School of Medical Sciences, 3-1-1, Maidashi, Higashi-ku, Fukuoka, Japan; 16 Department of Psychiatry, Kochi University School of Medicine, Kohasu, Okatoyocho, Nangoku-shi, Kochi, Japan; 17 Division of Psychiatry, Tokyo Metropolitan Matsuzawa Hospital, 2-1-1 Kamikitazawa, Setagaya-ku, Tokyo, Japan; 18 Department of Psychiatry, Geneva University, Department de Psychiatrie, 16-18, Bd de St Georges, 1205 Geneva, Switzerland

## Abstract

**Background:**

This study examines pathways to psychiatric care in Japan using the same method as the collaborative study carried out in 1991 under the auspices of the World Health Organization.

**Methods:**

Thirteen psychiatric facilities in Japan were involved. Of the 228 patients who contacted psychiatric facilities with any psychiatric illness, eighty four visiting psychiatric facilities for the first time were enrolled. Pathways to psychiatric care, delays from the onset of illness to treatment prior to reaching psychiatrists were surveyed.

**Results:**

Thirty three patients (39.4%) directly accessed mental health professionals, 32 patients (38.1%) reached them via general hospital, and 13 patients (15.5%) via private practitioners. The patients who consulted mental health professionals as their first carers took a longer time before consulting psychiatrists than the patients who consulted non-mental health professionals as their first carers. The patients who presented somatic symptoms as their main problem experienced longer delay from the onset of illness to psychiatric care than the patients who complained about depressive or anxiety symptoms. Prior to the visit to mental health professionals, patients were rarely informed about their diagnosis and did not receive appropriate treatments from their physicians. Private practitioners were more likely to prescribe psychotropics than physicians in general hospitals, but were less likely to inform their patients of their diagnosis.

**Conclusion:**

This first pathway to psychiatric care study in Japan demonstrated that referral pathway in Japan heavily relies on medical resources. The study indicates possible fields and gives indications, underlining the importance of improving skills and knowledge that will facilitate the recognition of psychiatric disorders presenting with somatic and depressive symptoms in the general health care system and by private practitioners.

## Background

An understanding of the way in which people seek care for mental disorders is important for planning mental health services, for the organization of training and for the organization of referrals to psychiatrists from other sources of health and social care. Goldberg and Huxley [1] proposed the 5 level model, which assumes that people with psychiatric problems start seeking care by consulting their general practitioner, who may refer them to psychiatric facilities. However, descriptive studies regarding this issue [2,3] demonstrated that people with psychiatric problems follow a variety of pathways before they reach mental health professionals, and that their pathways are influenced by various factors including conventions governing referral, relationships between mental health professionals and other sources of help, and the availability of and accessibility to mental health facilities and other helping agencies. Delays before people with mental illness receive appropriate care are also affected by several demographic factors, by diagnosis of the patients and by pathways they follow to reach psychiatrists.

The pathway study is a quick, useful and inexpensive method of studying help-seeking behavior of people with a mental illness. Pathway studies have been conducted in many countries but, to our knowledge, no study of pathways or people with mental health problems had been done in Japan. Yet, pathway studies in Japan are of particular interest because of the special features of the health system of Japan in which there are no general practitioners, and where patients are allowed to see any doctor of their choice.

## Methods

### Procedure

We have used the method developed for the World Health Organization multicenter pathway study [1], albeit with a shorter study period. All consecutive patients who visited mental health services for the first time within one calendar week between October 2003 and January 2004 were enrolled. A semi-structured interview based on an encounter form developed in the WHO collaborative study was conducted by mental health professionals with all the patients enrolled. We translated the encounter form and revised it slightly to adjust it to the situation in Japan. The encounter form served to record demographic data, the main problems presented by the patients, the source and type of care they received before they saw the mental health professional, and the length of time between the occurrence of their mental health problems and their contact with professional carers. The length of time at each step of care was also recorded. Psychiatric diagnoses according to ICD-10, and the total duration of illness were filled in by the psychiatrist in charge.

### The areas and participating centers

The participating centers were thirteen hospitals, of which seven were university hospitals, one a public general hospital and five mental hospitals. The study centers were in 12 cities across the nation. Each of them was the main provider of psychiatric services in each area (although psychiatric facilities may have also been located in their areas). The cities and their population, the number of psychiatric beds per 100,000 population and psychiatrists per 10,000 population are shown in Table 1.

**Table 1:** Participating centers

Name of institution	Type of institution	City	Population (thousand)	Psychiatric beds per 10,000 population	Psychiatric doctors per 100,000 population
Sapporo Medical University Hospital	UH	Sapporo	1,817	46	16
Iwate Medical University Hospital	UH	Morioka	288	50	15
Yokohama City University Medical Center	UH	Yokohama	3,381	16	8
Kansai Medical University Hospital	UH	Moriguchi	150	15	8
Nagasaki University Hospital	UH	Nagasaki	421	69	18
Kurume University Hospital	UH	Kurume	235	63	37
Fukuoka University Hospital	UH	Fukuoka	1,330	35	18
Wakkanai Municipal Hospital	GH	Wakkanai	44	23	9
Asai Hospital	MH	Togane	59	23	24
Sakuragaoka Memorial Hospital	MH	Tama	145	75	26
Zikei Hospital	MH	Okayama	621	49	24
Kochi Prefectural Geiyo Hospital	MH	Aki	21	72	28
Okawa Hospital	MH	Buzen	29	147	17

Whole nation			125,613	28.2	10.2

UH: University Hospital, GH: General Hospital, MH: Mental Hospital

The study was conducted under the auspices of the Japan Young Psychiatrist Organization (JYPO). The JYPO is a nationwide group of young psychiatrists aiming to promote academic development and networking in the field of psychiatry.

This study was approved by the institutional review boards of each participating center, and all subjects gave their written informed consent after having been given a full description of the study.

### Data analysis

The routes taken by individual patients were brought together to produce a "Pathway Diagram". The number of patients taking each step on the pathways was mapped onto the diagram along with and the delays occurring at each step. Delays were compared among major pathways, among different diagnostic groups and among presenting problems. We used median values when comparing delays because the distribution of delay was heavily skewed. Fisher's extract test was used for categorical data and Mann-Whitney non-parametric test was used for continuous data, using the SPSS version 15.0J software (SPSS Inc., Chicago, USA).

## Results

### Subject data

**Figure 1:**
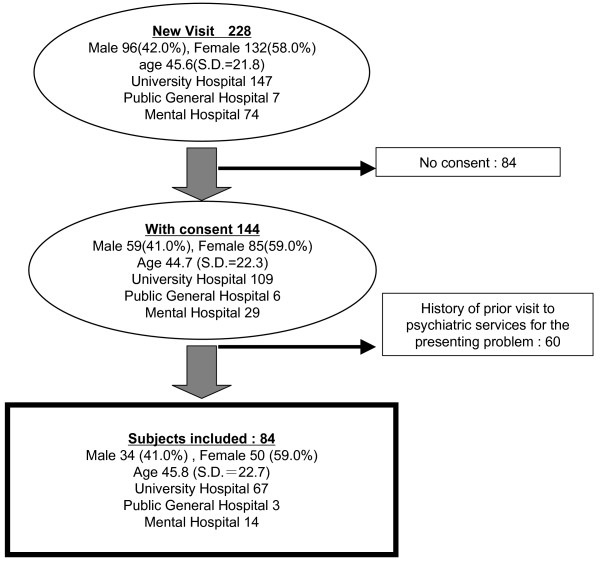
**Inclusion procedure and demographics of the subjects**.

Two hundred and twenty eight patients visited the participating centers for the first time during the study period. Written informed consent was obtained from 144 patients (68%), of which 84 patients (male 34: female 50) contacted psychiatric services for the first time because of the presenting problem (Figure 1). Sixty seven were seen at university hospitals, 3 at the public general hospital and 14 at mental hospitals. There were no significant differences in age and gender between subjects who consented and not consented to participate in the study.

### Main problem presented and diagnosis given by mental healthprofessionals

The main problems presented to the first carer are listed in Table 2. The most frequent presenting problems were somatic symptoms and depression (19 patients (22.8% each), followed by social problems (13 patients: 15.6%) and anxiety (12 patients: 14.5%). Distribution of diagnoses on ICD-10 is shown in Table 3. The most frequent diagnoses using ICD-10 criteria given by mental health professionals were mood disorders (F3) (21 patients: 25.0%), neurotic, stress-related and somatoform disorders (F4) (20 patients: 23.8%) and organic, including symptomatic, mental disorders (F0) (12 patients: 14.5%). Of 12 patients with F0 diagnosis, 7 patients were diagnosed as having dementia.

**Table 2:** Type of first carer and main problems presented

	Somatic	Depression	Social	Anxiety	Altered consciousness	Psychotic	Dementia related	Others	Total (%)
Mental Health Professionals	5	10	6	9	0	0	1	2	33 (39.3)
Other Carers	14	9	7	3	5	4	3	6	51 (60.7)

Total (%)	19 (22.8)	19 (22.8)	13 (15.6)	12 (14.5)	5 (6.0)	4 (4.8)	4 (4.8)	8 (9.5)	84 (100)

**Table 3:** Type of first carer and diagnosis given by mental health professionals

	F0	F2	F3	F4	F5	F6	Others	Total (%)
Direct Access to MHP	4	2	9	10	2	1	5	33 (39.4)
Indirect Access to MHP	8	2	12	10	3	2	14	51 (60.8)

Total (%)	12 (14.5)	4 (4.8)	21 (25.0)	20 (23.8)	5 (6.0)	3 (3.6)	19 (22.8)	84 (100)

Diagnosis based on ICD-10

F0: Organic, including symptomatic, mental disorders

F2: Schizophrenia, schizotypal and delusional disorders

F3: Mood disorders

F4: Neurotic, stress-related and somatoform disorders

F5: Behavioural syndromes associated with physiological disturbances and physical factors

F6: Disorders of adult personality and behaviour

MHP: Mental Health Professionals

### Pathway diagram

**Figure 2:**
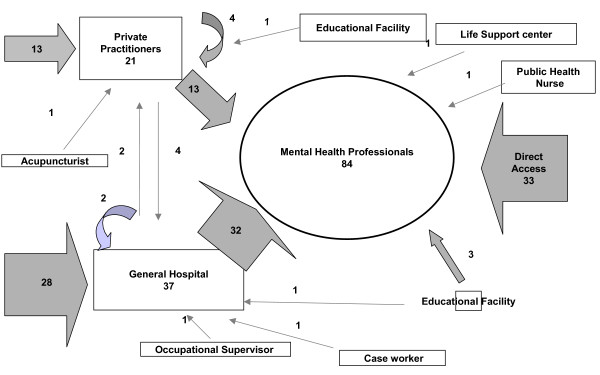
**The Pathway Diagram**. Figures indicate numbers of subjects who took each pathway or consulted each carer. Curved arrows indicate recursive pathways, where patients have gone from one to another of the same type of carer.

The sources of care utilized by the patients before they presented to psychiatric services are shown in Figure 2. Three major pathways were used – the direct pathway (contacting the mental health professional as first carer), the pathway via general hospitals ("GH pathway"), and the pathway via private practitioners ("PP pathway") comprise approximately 90% of the total subjects. Thirty three patients (39.4%) directly accessed mental health professionals, 32 patients (38.1%) reached them via GH pathway, and 13 patients (15.5%) via PP pathway. A small number of patients were referred from educational facilities (school teachers, university health center), a life support center and a public health nurse in the community.

### Delays to psychiatric care

The mean number of carers consulted prior to mental health professionals was 0.8 (S.D. = 0.9). The patients who first consulted general hospital saw average of 1.1 carers (S.D. = 0.4), and those who consulted private practitioners saw average of 1.5 carers (S.D. = 1.0) before they saw mental health professionals.

The distribution of delay has a long tail with progressively smaller numbers of patients having longer delays, inflating the mean delay to 87.4 weeks (S.D. = 284.8). Therefore, we adopted the same methodology as previous reports, and used median values. The median delays among total subjects and delays in main pathways are shown in Table 4. The median delay between the onset of the problem and contact with the first carer was two weeks; between the first carer and mental health professionals, zero week; and between the onset of the problem and consultation with mental health professionals were eight weeks.

**Table 4:** First carer, delays to psychiatric care and number of carers before patients reach mental health professionals

	Number of patients	Delays (median weeks)			Mean Number of carers prior to Mental Health Professionals (S.D.)
			
First carer		Onset to first carer	First carer to Mental Health Professionals	Onset to Mental Health Professionals	
Mental Health Professionals	33	8^a ^*,^b ^*	-	8	-
General Hospital Doctors	28	1^a ^*	1	3	1.1 (0.4)
Private Practitioners	13	4^b ^*	1	8.5	1.5 (1.0)

Total	84	2	0	8	0.8 (0.9)

a*, b*: p < 0.1 : median test

The median delay between the onset and consultation to the first carer was longest in direct pathway (8 weeks), and was significantly longer than other pathways (1 week in GH Pathway and 4 weeks in PP Pathway). The median delays between the first carer (general hospital doctor or Private Practitioner) and mental health professionals were 0 week. The median delays were not significantly different among three major pathways.

### Factors affecting the choice of pathway and delays

Table 5 shows relationship between presenting symptoms, choice of first carer and delays to psychiatric care. Patients with anxiety are more likely to go directly to mental health professionals, whereas patients with somatic symptoms were likely to firstly consult carers other than mental health professionals. Patients with depressive symptoms lie in between (p < 0.05).

**Table 5:** Main presented problems, first carer, delay and number of carers before patients reach mental health professionals

	First carer				Median delay (weeks)			Mean Number of Carers prior to Mental Health Professionals (S.D.)
			
	Mental Health Professionals	General Hospital Doctors	Private Practitioners	others	Onset to First Carer	First Carer to Mental Health Professionals	Onset to Mental Health Professionals	
Somatic (n = 19)	5^a ^*	8	3	3	3.0	1.0^b ^*	9.0	1.2^c ^* (1.0)
Depressive (n = 19)	10^a ^*	7	1	1	4.0	0	8.0	0.3 (0.5)
Anxiety (n = 12)	9^a ^*	3	0	0	2.5	0^b ^*	20.0	0.6^c ^* (0.8)

Total (n = 84)	33	27	13	11	2.0	0	8.0	0.8 (0.9)

a* : p < 0.05: Fisher's extract test, b* : p < 0.05: Median test, c* : p < 0.05: Mann-Whitney's U test

The patients with somatic symptoms take longer time and see larger number of carers before they reach mental health professionals, compared with those with anxiety symptoms. Age, gender, financial level, whether single or cohabitant, or past history of psychiatric disorder do not affect delays.

### Treatment by prior carers

Of 58 patients who were seen by non-psychiatric physicians, 37 patients were seen by general hospital doctors and 21 patients by private practitioners. We compared referral rate to mental health professionals, information about diagnosis given to patients, psychoeducation and medications given by hospital doctors and private practitioners.

#### (a) Referral to mental health professionals

Thirty two out of 37 patients who consulted general hospitals and 13 out of 21 patients who consulted private practitioners visited mental health professionals as their next carer. These patients are categorized into two groups: those who visited mental health professionals on their own decision (self-referral) and those who were referred by physicians (physician-referral). Twenty six out of 32 patients (81.3%) were referred by physician in general hospitals and 6 out of 13 (46.2%) by private practitioners (p < 0.05).

#### (b) Informed diagnoses and psychoeducation

Thirty one out of 58 patients were informed about their diagnosis (19 out of 28 at GH, 12 out of 21 at PP). Because of the small sample size, we limited statistical analysis to mood disorders and neurotic disorders. Accurate diagnoses were more likely to be told to patients by general hospital doctors than by private practitioners. Only to 3 out of 11 patients with depression seen by general hospital doctors were told their diagnosis and none was informed about diagnosis by private practitioners. In patients with anxiety, none out of 9 in general hospitals and only 1 out of 5 seen by private practitioners were told that they had neurotic disorders (Table 6).

**Table 6:** Referral rate and treatments by prior carers

				Psychiatric diagnosis		Treatment		
					
	Number of patients	Patients who visited MHPs as the next step	Patients referred to MHPs by prior carers	Informed to patients	Accurate diagnosis given	Benzodiazepines	Anti-depressants	Anti-psychotics
General Hospital Doctors				19	F3 3/11 F4 0/9	2	4	0

Private Practitioners	21	13 *	6 *	12	F3 0/4 F4 1/5	5	0	0

Total	58	45	32	31	F3 3/15 F4 1/14	7	4	0

F3: Mood disorders, F4: Neurotic, stress-related and somatoform disorders

MHPs: Mental Health Professionals

* P < 0.05 : Fisher's extract test

#### (c) Medications

Eleven out of 58 patients (19.0%) received psychotropic medications; 6 out of 37 (16.2%) by general hospital doctors, (hypnotics 2, antidepressants 4), and 5 out of 21 (23.8%) by private practitioners (anxiolytics only).

## Discussion

To our knowledge, this is the first multicenter study of pathways to psychiatric care in Japan. Our study provides a rough sketch of referral pathways to psychiatric care and some information about delays (and factors that influence them), treatments and psychoeducation given to the patients. Japan is unique in that it lacks general practitioners. We lack in training in general practice and most physicians in Japan are specialists in some field. Japan is also unique in that it employs free-referral medical system. That means, patients are allowed to see any hospital, any doctor of any subspecialty. Note that these two characteristics are quite important to understand the feature.

This diagnostic distribution is similar to those of previous pathway studies conducted in west European countries, including Spain[2], Italy[3] and United Kingdom[4].

The common presenting problems were somatic symptoms, depressive symptoms and anxiety symptoms. This is also similar to findings or previous pathways studies in developing and developed countries.

The pathway diagram demonstrates that, in Japan, 40% of all subjects have directly access to mental health professionals. Pathway studies have demonstrated that pathway to psychiatric care follow three patterns. The first pattern is dominated by the role of primary care physicians. Most patients first contact their general practitioner who refers them to mental health professionals. This pattern is seen in west and east European countries (Cantabria and Granada in Spain[2], Manchester in England[4], Benesov-Kromeriz in Czechoslovakia[5], Sofia in Bulgaria[5], Turgu Mures in Romania[5]), Aden in Yemen[2], Mexico City in Mexico[2], Havana in Cuba[2] and Sydney in Australia[6]. The second pattern is seen in Bali[7] and Ujung-Pandang (Indonesia)[2], Bangalore (India)[2], Harare (Zimbabwe)[2], Kwara (Nigeria)[8] and Rawalpindi (Pakistan)[2], where native healers play an important role in referral pathway. The third pattern is seen in Ankara (Turkey)[9], Lower-Silesia (Poland)[10], Verona (Italy)[3], where patients are allowed to see any carer of their choice and are likely to have directly access to mental health professionals. The nations of this pattern are likely to have larger proportion of patients who directly access mental health professionals. Our results are similar to those in countries with the third pattern. In Japan, patients are allowed to access any medical facilities of their choice, and patients with psychiatric problems prefer to see physicians in general hospitals rather than private practitioners. In contrast, in countries in which people are supposed to see general practitioners before they are seen by specialists (such as Spain[2], United Kingdom[4], Portugal[10], Czechoslovakia[2], and Australia[6], the pathway to mental health professionals via private practitioners is the most frequent and direct access is an exception.

Direct access to mental health professionals has both advantages and disadvantages. In the Goldberg and Huxley model[1], general practitioners are expected to function as "gate keepers" to apportion patients with a more severe form of illness to higher levels of specialization by keeping milder patients at lower levels. This gate-keeping role is supposed to enable psychiatrists to concentrate on patients with more severe forms of illness. Direct accessibility to mental health professionals may lead to wasteful use of the time of highly specialized professionals who would treat milder forms of illness which could be very well done by general practitioners. Such an arrangement would thus increase the cost of care and deteriorate medical economical efficiency. On the other hands, direct accessibility to mental health professionals may shorten the total delay between the onset of symptom and arrival at mental health professionals for patients who may have milder symptoms in the beginning of their illness but who do not recover as well when treated by general practitioners.

There are two types of delay in reaching psychiatric care. The first type of delay is the delay between the onset of the problem and the contact with the first carer. The length of this type of delay depends on the process of patients' recognition of the problem and their readiness to seek help. The second type of delay is that caused by contacting a carer who is not a mental health professional. This delay depends on the time that carers take before they recognize a patient's problem or discover that their treatment of that problem was not successful, which makes them refer the patient to a mental health professional.

Our study showed that the delay between the onset of the symptom and contact to mental health professionals was the shortest among the patients who firstly accessed general hospitals (3 median weeks), compared with those among the patients who accessed private practitioners or directly accessed mental health professionals (8 median weeks, respectively). Patients tends to access general hospital or private practitioners more quickly than they access mental health professionals (p < 0.1). However, the advantage of early visit to the first carer is offset by the delay between the first carer and the mental health professionals; therefore total delay in this pathway becomes not significantly different among GH pathway, PP pathway and direct access. This is so for patients who did not improve under treatment by the non-mental health professionals, or were not immediately recognized as having a mental illness; all others – who reacted well to treatment or improved spontaneously – were better off having contacted general health facilities because they avoided stigmatization.

Physicians working in general hospitals refer their patients more quickly to mental health professionals than private practitioners. This may be because physicians in general hospitals are more specialized in their field of interest, which might enhance quicker referral compared with private practitioners, who are supposed to be more ''general'' in their practice. Compared with general hospital doctors, private practitioners are more likely to prescribe psychotropics and to give psychiatric diagnosis, although somewhat inappropriately.

The patients who presented somatic symptoms as their main problem experienced longer delay than patients who complained about psychiatric symptoms. This is similar to findings of studies in other countries. The reason for this finding may be that many such patients do not regard their problem as psychiatric symptoms and that they request their physician to carry out time-consuming physical examinations, and because physicians might think that they need to take their time for physical examinations to rule out physical illness.

Compared to patients with anxiety, patients with depressive symptoms are more likely to first seek care by contacting non-mental health professionals. Prior pathway studies suggest that psychotic feature lead to shorter delays. Our study didn't support this, presumably due to small sample size.

Overall, patients access the first carer within a few weeks and then reach mental health professionals within one median week. These delays are as short as those in Spain[2], Cuba[2] and Turkey[9], and one of the shortest among pathway studies to date. This may be because at the number of psychiatrists per capita in Japan is much higher than those in countries in prior studies, as well as because patients are allowed to see any doctor or psychiatrist of their choice.

Compared with prior pathway studies, our study is unique in that we surveyed whether patients were told what their diagnosis was and explored care given to patients prior to the visit of mental health professionals. In our country, patients were rarely told their diagnosis and rarely received appropriate treatments from non-psychiatrists. Private practitioners were more likely to prescribe psychotropics compared with physicians in general hospitals, but were less likely to tell patients their diagnosis.

Our study has some limitations. First, small sample size makes it difficult to evaluate the effect of variation in diagnoses and characteristics of participating facilities. Second, participating centers were biased in their characteristics and locations. Psychiatric outpatient clinics (without wards) were not included in our study. The distribution of the diagnoses may have been influenced by unevenness in numbers and types of patients seen in the participating centers. Third, information gathered in this study is based on the willingness of patients to acknowledge their previous source of care. Thus, patients may have been reluctant to disclose contacts with carers (such as religious or traditional healers) or deny previous psychiatric treatment. Finally, as mentioned in previous reports, this study gives no account of those who do not reach mental health services.

Despite these limitations, this study is noteworthy in that this is the first multicenter study on pathway to psychiatric care in Japan. We hope that this study will generate hypotheses and studies focused on ways of improving the mental health care system in Japan.

## Conclusion

The first pathway to psychiatric care study in Japan demonstrated that referral pathway in Japan heavily relies on medical resources. Approximately 40% of the patients directly access mental health professionals, another 40% via general hospital, and 15% via private practitioners. The study indicates importance of improving skills and knowledge that will facilitate the recognition of psychiatric disorders presenting with somatic and depressive symptoms especially among private practitioners.

## Competing interests

The authors declare that they have no competing interests.

## Authors' contributions

DF, NH and YMK had full access to the data and performed the statistical analysis. DF designed the study and drafted the manuscript. NH helped drafting the manuscript. KO managed the data. GO and MT participated in study design. AN conceived the study and participated in coordination of the study. RS, TK, ET, KY, TM, HT, SS, HI, YW, TU, IM were research directors of each participating center and played essential role in data acquisition. KT participated in data management. NS conceived the study, critically revised the manuscript for important intellectual content. All authors read and approved the final manuscript.
